# Social Support and Technology Use and Their Association With Mental and Physical Health During the COVID-19 Pandemic Among Asian Americans: The COMPASS Cross-sectional Study

**DOI:** 10.2196/35748

**Published:** 2023-01-23

**Authors:** Linda G Park, Oanh L Meyer, Marcelle M Dougan, Bethany Golden, Kevin Ta, Bora Nam, Janice Y Tsoh, Marian Tzuang, Van M Ta Park

**Affiliations:** 1 Department of Community Health Systems School of Nursing University of California, San Francisco San Francisco, CA United States; 2 Department of Neurology School of Medicine University of California, Davis Sacramento, CA United States; 3 Department of Public Health and Recreation San José State University San Jose, CA United States; 4 Department of Family Health Care Nursing School of Nursing University of California, San Francisco San Francisco, CA United States; 5 Department of Psychiatry and Behavioral Sciences School of Medicine University of California, San Francisco San Francisco, CA United States; 6 Asian American Research Center on Health University of California, San Francisco San Francisco, CA United States; 7 Multiethnic Health Equity Research Center University of California, San Francisco San Francisco, CA United States

**Keywords:** health disparities, mental health, depression, anxiety, social support technology, COVID-19, pandemic, disparity, support, technology, physical health, race, survey, population, discrimination, outcome, AAPI

## Abstract

**Background:**

The global COVID-19 pandemic disproportionately affected Asian Americans and Pacific Islanders (AAPIs) and revealed significant health disparities with reports of increased discrimination and xenophobia. Among AAPIs, the pandemic exacerbated their social, linguistic, and geographic isolation. Social support may be especially important for AAPIs given the salience of collectivism as a cultural value. Another mechanism for support among AAPIs was technology use, as it is generally widespread among this population. However, older adults may not perceive the same benefits.

**Objective:**

We examined social support and technology use and their relationships with mental and physical health outcomes through the COVID-19 pandemic among AAPIs.

**Methods:**

Data were drawn from the COVID-19 Effects on the Mental and Physical Health of AAPI Survey Study (COMPASS) for the time period of October 2020 to February 2021. COMPASS was a cross-sectional, multilingual, national survey conducted online, by phone, and in person with AAPI adults who were ≥18 years of age, in collaboration with academic and community partners in the United States. Data were analyzed using multivariable linear regression using the outcome variables of mental and physical health with various predictors such as social support and technology use. We tested for interactions specific to age and ethnicity.

**Results:**

Among 4631 AAPIs (mean age 45.9, SD 16.3 years; 2992/4631, 63.1% female), we found that (1) increased social support was associated with better physical health, (2) total social support was positively associated with better mental health, (3) higher technology use was associated with poorer mental health and inversely associated with poorer physical health, (4) the association of technology use with mental health was weaker among those with low social support (vs those with high social support), (5) adults younger than 60 years old (vs ≥60 years old) were more negatively affected with social support and mental health, and (6) Korean Americans appeared to be a high-risk group for poor physical health with increased technology use.

**Conclusions:**

Our paper identified mental and physical health needs along with supportive therapies observed among AAPIs during the pandemic. Future research on how social support can be leveraged, especially among AAPIs younger than 60 years old, and how various types of technology are being utilized are important to guide the recovery efforts to address both mental and physical disparities across communities as a result of the COVID-19 pandemic.

## Introduction

The COVID-19 pandemic has had widespread health, social, and economic implications that the world has not experienced in modern history. Asian Americans and Pacific Islanders (AAPIs) are the most rapidly growing and heterogeneous racial group in the United States, with very limited research participation [1-3]. It has been brought to the forefront that significant health disparities, socioeconomic inequalities, and discrimination and xenophobia exist for AAPIs, both prior to and due to COVID-19 [4-6]. As a result of policies (eg, shelter-in-place [SIP], social distancing) that were implemented, persons and communities of color and those who are socially and technologically isolated were among the most vulnerable in terms of the adverse effects of COVID-19 [7,8]. Older AAPIs are especially vulnerable and more likely to be disproportionately affected by COVID-19 policies due to higher rates of poverty, lower educational attainment, limited English proficiency [9], and less access to digital technology [9,10] (eg, internet) or lower digital literacy [11], which has been an integral tool for social, educational, and health purposes during this pandemic [12].

The pandemic has exacerbated the social, linguistic, and geographic isolation of AAPIs [13-15]. Social isolation and the need for support are prevalent among older AAPIs whose English language proficiency is limited. Social support may be especially relevant for AAPIs given the salience of collectivism as a cultural value. Research has shown that having social connections and supportive resources can buffer the effects of social isolation and is a strong determinant of health and well-being [13,16]. Having emotional support lowers the adverse effects of stress, increases emotions that are positive, and decreases the duration and intensity of emotions that are negative [17]. Studies have shown the positive buffering effects of social support on health and well-being in AAPIs for culture and ethnicity-related stressors (eg, discrimination) [18,19].

The use of digital technology became a necessity to communicate and sustain daily activities during the COVID-19 pandemic [20,21]. Digital technology refers to a smartphone, computer, internet service, tablet, and television with cable. English-speaking Asian Americans had the highest technology adoption rates in the United States compared with other racial groups, with 62% of Asian Americans having access to an internet-connecting device such as a router (compared with 47% of Whites, 48% of Blacks, and 45% of Hispanics) and 97% owning a smartphone [22]. Although smartphone ownership and internet use have grown in popularity over the past decade, with the largest adoption rates among older adults, research is still lacking among older AAPIs, specifically those who have limited English or digital literacy and may have wide variations in income.

SIP and social distancing made the use of digital technology crucial for numerous reasons, ranging from health maintenance to social support to information gathering. Social media, for example, can provide individuals with opportunities for widening social circles, maintaining social ties, and increasing feelings of inclusion [23,24]. In examining digital technology, it is imperative to consider the ways in which technology is utilized, as different digital technology uses have different effects on mental and physical health. Nonpassive use of social media, such as interacting with others and posting online, is related with better well-being through social support [25,26]. On the other hand, passive use of digital technology, such as surfing the web, reading the news, and watching movies, is typically related to poorer well-being, as these activities require no interactions between people [27]. Additionally, consistent use of technology can create unhealthy habits (such as psychological dependence and addiction to technology use), which have been associated with higher perceived social isolation [28]. As a result of the pandemic, the use of digital technology became the only source of connection and social support available for many [29]. People have transitioned to Zoom and other video conferencing services for both work and maintaining personal relationships [30]. Patients have utilized telemedicine more than ever to receive care via phone or video [31,32].

The COVID-19 pandemic has greatly driven technology’s role as a source of social support [33-35]. For older adults, the use of digital technology has facilitated access to health resources, with the percentage of older adults who had ever participated in a telehealth visit rising sharply from 4% in 2019 to 30% in 2020 [36]. Similarly, the use of information and communication technology among older adults has facilitated different levels of social support such as making connections and emotional support [37]. Despite these findings, there is a lack of research on how AAPIs, as well as older AAPIs, utilize digital technology as a form of social support as a buffering effect alongside stress and isolation.

Given the importance of social support and technology use in the era of a global COVID-19 pandemic, we sought to collect data in a national survey (COVID-19 Effects on the Mental and Physical Health of Asian Americans and Pacific Islanders Survey Study [COMPASS]) that represent the experiences of AAPIs. The objective of this paper was to examine social support and use of digital technology and their associations with mental and physical health during the COVID-19 pandemic among AAPIs. This study sought to inform mental health providers, public health officials, and community partners about the role of social support in mental and physical health during the pandemic as well as the impact of the increased use of technology in AAPI communities.

## Methods

### Recruitment

There were 5418 participants who completed the survey from October 24, 2020, to April 10, 2021. The COMPASS survey was distributed through community organizations that serve AAPIs, personal and professional networks via email and listservs, social media, flyers, and directed ethnic media. The Collaborative Approach for Asian Americans and Pacific Islanders Research and Education (CARE) research recruitment registry [38,39] was also used to recruit via email. A US $10 gift card was offered upon survey completion. To encourage participation, the survey could be completed online on the COMPASS website [40], by phone, and limited in-person survey administration assistance by COMPASS’ staff and community partners. Of the participants, 86.0% (4563/5304) completed the survey independently, and 14.0% (741/5304) had assistance from family, friends, or research staff. To maximize the use of available data, we excluded 787 participants who did not provide information on social support (n=729) or mental health (n=58) from the mental health analyses. Participants who did not provide information on technology use (n=106) or on their physical health (n=224) were further excluded from the analyses including these variables.

### Ethical Approval

We obtained human subject research approval from the Institutional Review Board at University of California, San Francisco (Protocol #20-31925), and informed consent was obtained from the participants prior to completing the COMPASS cross-sectional online survey.

### Study Eligibility and Procedures

Eligible participants were required to be 18 years of age or older; live within the United States; self-identify ethnically as an AAPI (full or combined with another race or ethnicity); and comprehend written English or Chinese (traditional or simplified Chinese), Korean, Samoan, or Vietnamese. The World Health Organization’s process of adapting instruments [41], including backward and forward translation, was used to guide the multilanguage survey’s scale and material development. Most participants completed the survey in English (3503/5418, 64.5%); however, there were 4.8% (261/5418) surveys completed in simplified Chinese, 4.7% (253/5418) completed in traditional Chinese, 12.7% (686/5418) completed in Korean, 3.5% (189/5418) completed in Samoan, and 9.7% (526/5418) completed in Vietnamese.

### Measures

Mental health was assessed using the Patient Health Questionnaire-4 (PHQ-4) scale for depressive and anxiety symptoms [42]. The PHQ-4 consists of 2 items that measure depressive symptoms [43] and 2 items that measure anxiety symptoms [44]. Each item had response options ranging from 0 to 3, and a PHQ-4 score was created by summing the 4 items (range: 0-12), with higher scores indicating more severe symptoms. Mental health was measured by summing the responses across each of the 4 questions that generated a PHQ-4 score. PHQ-4 has demonstrated acceptable reliability (Cronbach alpha=.82) and construct validity [45] in the general population. In our population, the Cronbach alpha was .90, suggesting acceptable reliability. Scores of 0 to 2 indicated normal, 3 to 5 indicated mild, 6 to 8 indicated moderate, and 9 to 12 indicated severe depression or anxiety. In descriptive analyses by age, we also examined total PHQ-4 score as a categorical variable by comparing those older and younger than 60 years.

General perceived physical health was measured by asking participants to indicate their health “today” using the EuroQol EQ-5D [46,47]. The scale ranges from 0 (worst) to 100 (the best health you can imagine). EQ-5D has been translated and validated in many languages (including Chinese, Korean, and Vietnamese) [48].

Social support was measured using the 6-item Social Support Inventory from the National Latino Asian American Study. Total social support was examined as an average across 6 items: 3 related to family support and 3 related to friend support. The questions included: “How often do you talk on the phone or get together with relatives/friends?”; “How much can you rely on relatives/friends for help with a serious problem?”; and “How much can you open up to family/friends and talk about worries?” Corresponding points were added for each level of support (from 1 to 5 or 1 to 4, as appropriate), with the responses “Refused” and “Don’t know” coded as missing. These were then reverse scored, such that higher values corresponded with more social support. Average scores were computed by summing all the individual items and dividing by 6. The Cronbach alpha across all 6 measures was .78.

The COMPASS team created a survey to assess patterns of technology use during the COVID-19 pandemic (Multimedia Appendix 1). The questions related to this study included “Did your use of technology increase during the COVID-19 crisis?” Response options were in the following 5 categories: “Did not increase,” “Increased by 1-2 hours/day,” “Increased by 3-4 hours/day,” “Increased by 5-6 hours/day,” and “Increased by 7+ hours/day.” As we were interested primarily in the effects of increased technology use, we combined the 2 lowest categories of “Did not increase” and “Increased by 1-2 hours/day.”

For sociodemographic and other COVID-19–related characteristics, participants were asked about their year of birth, sex, sexual orientation, race, ethnic or cultural group, marital status, country of birth, education, employment status, and annual household income in 2019. The question asking about the length of SIP orders was developed by the COMPASS team and asked: “How long was the shelter-in-place (or stay-at-home) order at where you live?” The response options included 0 (no order), 1 (<1 month), 2 (1-2 months), and 3 (≥2 months). The question about perceived severity of COVID-19 was also developed by the COMPASS team and asked the participants: “How would you rate the severity of COVID-19 outbreak where you live in comparison to other locations in the U.S.?” Response options were 1 (a lot less severe than most other places in the U.S.), 2 (somewhat less severe), 3 (about the same), 4 (somewhat more severe), and 5 (a lot more severe). The effect of COVID-19 on family income or employment was measured by 1 of the items from the Coronavirus Impact Scale [49]. Participants were asked to rate how much the COVID-19 pandemic has changed their family income or employment. Response options included 0 (No change), 1 (Mild. Small change; able to meet all needs and pay bills), 2 (Moderate. Having to make cuts but able to meet basic needs and pay bills), and 3 (Severe. Unable to meet basic needs and/or pay bills).

### Statistical Analysis

This study analyzed 2 outcome variables, specifically mental health and physical health. Mental health was measured using the PHQ-4 score (sum of 4 items) that was modeled as a continuous variable using multivariable linear regression analysis, with higher numbers indicating weaker mental health, which is consistent with how previous researchers have analyzed it [42,45]. A total of 3 or more for the first 2 PHQ-4 items is indicative of anxiety, and a total of 3 or more for the last 2 items is indicative of depression. Elevated depression or anxiety was indicated by a total of 3 or more across all 4 items. Physical health was also modeled as a continuous variable using multivariable linear regression analysis, with higher numbers indicating better physical health [46,47]. For the main independent variables of interest, social support was modeled as a continuous variable, while technology use was modeled as a categorical variable.

Descriptive statistics were used to report the sociodemographic characteristics of the sample. In the multivariable linear regression models, we adjusted for sociodemographic variables (age, cultural group, sex, country of birth, language proficiency, marital status, employment, education, income) since these variables are known to influence physical and mental health outcomes. We also adjusted for COVID-19–related variables (change in family income, length of SIP, perceived severity of COVID-19, and region) as they were contextual in the era of the pandemic and hypothesized to influence health outcomes. We examined multicollinearity using the variance inflation factor (VIF). The VIF for each of the variables in all physical and mental health models was <5, suggesting little or no multicollinearity between the variables. 

To model interactions, we selected hypothesized modifiers of the relationship between our main independent variables and outcomes, specifically age and ethnicity. Age was categorized as a dichotomous variable representing age 60 years or older versus younger than 60 years old based on the age that is considered elderly by international standards [50]. For ethnicity, we selected the 3 largest groups to represent adequate sample sizes for interaction analyses: Ethnic Chinese (n=1560), Korean (n=1007), and Vietnamese (n=841). We examined interactions with age and ethnicity to detect age-related and ethnic or cultural differences, respectively, that may be related to all of the variables of interest (ie, social support, mental and physical health, technology use). We modeled interactions by multiplying the independent variable with the categorical variable (age and ethnicity) and used the Wald test for statistical significance. Tests for changes in technology use were conducted by modeling increase in technology use as a categorical variable with 5 categories ranging from decreased or stayed the same to ≥7–hour per-day increase, with decreased or staying the same as the reference group and using the Wald test for statistical significance. We also tested for a linear trend in technology use using an ordinal variable for technology use and assessing whether the *P* value was significant.

We sought to test the joint effect of social support and technology use on mental and physical health by modeling social support as both a continuous variable and a dichotomous variable (categorized below and above the median) and technology use as an ordinal variable. For the purposes of interpretability of the results, the interaction between social support and technology use was described using social support as a dichotomous variable (at or above the median vs below) and modeling technology use as an ordinal variable. We then used *P* values to assess whether the interaction was statistically significant for technology and high versus low social support.

All statistical tests were 2-sided with alphas for statistical significance set at ≤.05 for all tests. Statistical analyses were conducted using SAS software [51].

## Results

### Participant Characteristics

Table 1 describes the participant characteristics (n=4631). The major cultural groups were ethnic Chinese, Korean, and Vietnamese. Participants were mostly female (2992/4631, 63.1%). The mean age of participants was 45.9 (SD 16.3) years. Overall, the majority of participants were foreign-born (2976/4631, 64.3%); these foreign-born participants had lived in the United States an average of 24.7 (SD 15.1) years. The mean physical health score was 78.1 (SD 14.9) on a scale of 0 to 100.

Overall, based on the PHQ-4, 37.7% (1745/4631) of participants had elevated depression or anxiety. The prevalence of mild depression among those older than 60 years was lower than in those younger than 60 years (212/1044, 20.3% vs 1030/3587, 28.7%, respectively). Similarly, the prevalence of moderate (32/1044, 3.1% vs 320/3587, 8.9%) and severe depression (14/1044, 1.4% vs 137/3587, 3.8%) was lower among those aged 60 years and older compared with those younger than 60 years, respectively.

**Table 1:** Sociodemographic and background characteristics for all participants (n=4631).

Characteristics		Results
**Cultural group, n (%)**		
	Asian-Indian	282 (6.1)
	Ethnic Chinese^a^	1560 (33.7)
	Filipino	165 (3.6)
	Hmong	98 (2.1)
	Japanese	202 (4.4)
	Korean	1007 (21.7)
	Native Hawaiian/Pacific Islander	246 (5.3)
	Vietnamese	841 (18.2)
	Other/mixed	232 (5.0)
**Sex, n (%)**		
	Female	2992 (63.1)
	Male	1669 (36.0)
	Other/decline to answer	40 (0.9)
**Sexual orientation, n (%)**		
	Heterosexual	4247 (91.7)
	Not heterosexual	209 (4.5)
	Decline to answer	175 (3.8)
Age (years), mean (SD)		45.9 (16.3)
Age (years), range		18-97
**Age (years), n (%)**		
	18-30	967 (20.9)
	30-39	797 (17.2)
	40-49	825 (17.8)
	50-59	998 (21.6)
	≥60	1004 (22.5)
**Country of birth, n (%)**		
	United States	1593 (34.4)
	Foreign country	2976 (64.3)
	Other	1 (0.02)
	Don’t know	61 (1.3)
**Limited English proficiency, n (%)**		
	Yes	1092 (23.6)
	No	3539 (76.4)
**Marital status, n (%)**		
	Single	1208 (26.1)
	Married/living with partner	3062 (66.1)
	Separated/divorced/widowed	326 (7.0)
	Declined to answer	35 (0.8)
**Employment status, n (%)**		
	Full time	2147 (46.4)
	Part time	774 (16.7)
	Homemaker	372 (8.0)
	Unemployed	507 (11.0)
	Retired	541 (11.7)
	Other/declined to answer	290 (6.3)
**Education, n (%)**		
	High school or less	801 (17.5)
	Some college or technical school	509 (11.1)
	Bachelor’s degree	1648 (36.1)
	Master’s degree or higher	1613 (35.3)
**Household income (US $), n (%)**		
	≤25,000	869 (18.8)
	>25,000-75,000	1230 (26.6)
	>75,000-150,000	1135 (24.5)
	>150,000	914 (19.7)
	Declined to state	483 (10.4)
**Length of SIP^b^ order (n=5309, 17 missing responses), n (%)**		
	No order	438 (9.5)
	<1 month	255 (5.5)
	1 to <2 months	514 (11.1)
	2 to <3 months	520 (11.3)
	≥3 months	2545 (55.2)
	Don't know	342 (7.4)
**The severity of COVID-19 where you live (n=4613, 23 missing responses), n (%)**		
	A lot less	512 (11.1)
	Somewhat less	794 (17.2)
	About the same	984 (21.3)
	Somewhat more	1337 (29.0)
	A lot more	986 (21.4)
**Technology use due to COVID-19 (n=4525, 106 missing responses)**		
	Decreased or stayed the same	913 (20.2)
	Increased by 1-2 hours/day	1364 (30.1)
	Increased by 3-4 hours/day	1416 (31.3)
	Increased by 5-6 hours/day	547 (12.1)
	Increased by ≥7 hours/day	285 (6.3)
Social support score, mean (SD)		3.01 (0.71)
Social support score, range		1-4.3
PHQ-4^c^, range		0-12
**PHQ-4, mean (SD)**		
	Entire sample	2.23 (2.62)
	<60 years old	2.48 (2.71)
	≥60 years old	1.36 (2.10)
Physical health score, range		0-100
**Physical health score, mean (SD)**		
	Entire sample	78.1 (14.9)
	<60 years old	78.2 (15.0)
	≥60 years old	77.6 (14.8)
Missing		236^d^

^a^Ethnic Chinese includes mainland Chinese, Hongkonger, Taiwanese, and Huaren.

^b^SIP: shelter in place.

^c^PHQ-4: Patient Health Questionnaire-4.

^d^Additional 12 missing technology use in addition to physical health; means and SD are similar for the available data.

### Missing Data

There were up to 13% missing data on social support; however, only 5.4% of the physical health data were missing, and 1.6% of the mental health data were missing. Participants who were missing complete information on social support were similar to participants who were included (ie, age, sex, ethnic group) but more likely to have limited English proficiency (495/4072, 17.4% vs 234/1346, 12.2%; *P*<.001).

### Social Support and Mental Health

Higher total social support was associated with better mental health. Total social support was inversely associated with PHQ-4 score in the crude and adjusted analyses. In the crude analysis, a 1-unit increase in average total support was associated with a lower total PHQ-4 score (=–0.51; 95% CI –0.61 to –0.40; *P*<.001). The association was attenuated in the adjusted analysis but remained significant: –0.35 (95% CI –0.45 to –0.25; *P*<.001; Table 2). We found evidence of a statistically significant interaction between age and social support in relation to mental health (*P*=.01), comparing those who were aged 60 years and older to those younger than 60 years old. In the stratified analyses by age, the association with total support was significantly stronger for those aged less than 60 years compared with those aged 60 years or older: =–0.563 (95% CI –0.69 to 0.44; *P*<.001) and =–0.17 (95% CI –0.34 to –0.01; *P*=.049), respectively (*P_interaction_*=.008; Table 2).

**Table 2:** Social support in relation to mental health and physical health, overall and stratified by age, for all participants.

Social support	Crude					Adjusted^a^			
	Overall (n=4631), beta (95% CI)	*P* value	Overall (n=4631), beta (95% CI)	*P* value	18-60 years old (n=3587), beta (95% CI)		*P* value	≥60 years old (n=1044), beta (95% CI)	*P* value
Total support for mental health^b^	–0.51 (–0.61 to –0.40)^c^	<.001	–0.35 (–0.45 to –0.25)^d^	<.001	–0.56 (–0.69 to –0.44)^e^		<.001	–0.17 (–0.34 to –0.01)^f^	.049
Total support for physical support^g^	2.94 (2.32 to 3.56)^h^	<.001	2.81 (2.18 to 3.45)^i^	<.001	3.33 (2.60 to 4.07)^j^		<.001	0.95 (–0.36 to 2.26)^k^	.15

^a^Adjusted for age (18-30, 30-39, 40-49, 50-59, ≥60+ years), sex (male, female, other/don’t know), marital status (single, married/living with partner, separate/divorced/widowed, declined to answer), employment status (full time, part-time home maker, unemployed, retired, other/declined to answer), cultural group (Asian Indian, ethnic Chinese, Filipino, Hmong, Japanese, Korean, Native Hawaiian and Pacific Islanders, Vietnamese, other/mixed), United States region (Midwest, Northeast, South, West), severity of COVID-19 in participant’s location relative to the rest of the United States (a lot less, somewhat less, about the same, somewhat more, a lot more), annual household income (US $; ≤25,000, 25,001-75,000, 75,001-150,000, ≥150,001, declined to answer), education (high school or less, some college or technical school, bachelor’s degree, master’s degree or higher), length of shelter in place (no order, <1 month, 1 month to <2 months, 2-3 months, don’t know), change in family income (no change, mild, moderate, severe), English proficiency (limited, not limited), country of birth (United States, outside the United States).

^b^*P_interaction_* for age x social support in relation to mental health=.008.

^c^Model *R^2^*=0.02.

^d^Model *R^2^*=0.21.

^e^Model *R^2^*=0.14.

^f^Model *R^2^*=0.12.

^g^*P_interaction_* for age x social support in relation to physical health=.01.

^h^Model *R^2^*=0.02.

^i^Model *R^2^*=0.04.

^j^Model *R^2^*=0.05.

^k^Model *R^2^*=0.06.

### Social Support and Physical Health

Social support was positively associated with the physical health score. In the crude analysis, increased social support was associated with higher physical health scores. For every unit increase in mean total support score, the average physical health score was 2.94 (95% CI –2.32 to 3.56, *P*<.001). This association persisted in the adjusted analysis: A 1-unit increase in mean total support was associated with a 2.81 (95% CI 2.18 to 3.45; *P*<.001) increase in the physical health score (Table 2). This association appeared limited (*P_interaction_*=.01) to those aged less than 60 years of age (=3.33, 95% CI 2.60 to 4.07; *P*<.001). The association between social support and physical health was not significant among those aged 60 years and older (Table 2).

### Technology Use and Mental Health

Technology use at each level was associated with an increase in total PHQ-4 score in the crude models, an association that persisted in the adjusted models. Compared with those for whom technology use decreased or stayed the same during the pandemic, after adjusting for covariates, those whose technology increased by 7 hours or more per day had a 1.32-point (95% CI 1.00 to 1.64; *P* for the trend<.001) higher PHQ-4 score (Table 3). The differences by age group and ethnicity were not statistically significant (data not shown). Table S1 in Multimedia Appendix 2 presents the baseline technology use between ethnic groups.

**Table 3:** Mental health in relation to amount of technology use for all the participants.

Mental health	Crude^a^, beta (95% CI)	*P* value	Adjusted^b,c^, beta (95% CI)	*P* value
Decreased or stayed the same	Reference	N/A^d^	Reference	N/A
1-2 hour/day increase	0.33 (0.12 to 0.54)	.002	0.19 (–0.01 to 0.38)	.055
3-4 hour/day increase	1.11 (0.91 to 1.32)	<.001	0.63 (0.43 to 0.83)	<.001
5-6 hour/day increase	1.61 (1.34 to 1.87)	<.001	0.86 (0.61 to 1.12)	<.001
≥7 hour/day increase	2.15 (1.82 to 2.49)	<.001	1.32 (1.00 to 1.64)	<.001

^a^Model *R^2^*=0.06; *P* for trend<.001.

^b^Adjusted for the same variables as in Table 2.

^c^Model *R^2^*=0.22; *P* for trend<.001.

^d^N/A: not applicable.

### Technology Use and Physical Health

Technology use was inversely associated with physical health. Compared with the reference group, those whose use increased by 7 hours or more a day reported a –3.48 (95% CI –5.50 to –1.46; *P* for the trend<.001) physical health score in the crude model. There was a statistically significant interaction by cultural group, with Koreans (*P* for the trend<.001) having significantly lower health scores for 7 or more hours of technology use daily compared with the same or decreased use; this was not true for ethnic Chinese (*P* for the trend=.48) or Vietnamese (*P* for the trend=.16; Table 4). The interaction by age was not significant (data not shown).

**Table 4:** Physical health in relation to technology use, overall and stratified by cultural group, for all participants.

Physical health	Crude^a^			Adjusted^b^							
	Overall, beta (95% CI)	*P* value	Overall^c^, beta (95% CI)		*P* value	Ethnic Chinese^d,e^, beta (95% CI)	*P* value	Korean^d,f^, beta (95% CI)	*P* value	Vietnamese^d,g^, beta (95% CI)	*P* value
Decreased or stayed the same	Reference	N/A^d^	Reference		N/A	Reference	N/A	Reference	N/A	Reference	N/A
1-2 hour/day increase	–0.80 (–2.05 to 0.44)	.21	–1.22 (–2.49 to 0.05)		.06	0.44 (–2.65 to 1.77)	.69	–1.49 (–4.94 to 1.96)	.40	0.91 (–1.54 to 3.36)	.56
3-4 hour/day increase	–2.18 (–3.41 to –0.94)	<.001	–2.55 (–3.85 to –1.25)		<.001	–0.83 (–3.03 to 1.37)	.46	–2.82 (–6.28 to 0.64)	.11	–1.68 (–4.37 to 1.00)	.22
5-6 hour/day increase	–2.87 (–4.46 to –1.28)	<.001	–3.43 (–5.10 to –1.76)		<.001	–1.58 (–4.24 to 1.09)	.25	–7.75 (–12.21 to –3.29)	.001	0.62 (–3.44 to 4.69)	.76
≥7 hour/day increase	–3.48 (–5.50 to –1.46)	<.001	–4.02 (–6.10 to –1.94)		<.001	–0.30 (–3.69 to 3.10)	.87	–9.39 (–15.02 to –3.76)	.001	–3.82 (–8.60 to 0.96)	.12

^a^Model *R^2^*=0.02; *P* for trend<.001.

^b^Adjusted for the same variables as Table 2 except analyses stratified by cultural group

^c^Model *R^2^*=0.04; *P* for trend<.001.

^d^P*_interaction_* for technology x cultural group in relation to physical health=.007

^e^Model *R^2^*=0.03; *P* for trend=.48.

^f^Model *R^2^*=0.04; *P* for trend<.001.

^g^Model *R^2^*=0.08; *P* for trend=.16.

### Social Support and Technology Use in Relation to Mental and Physical Health

**Figure 1:**
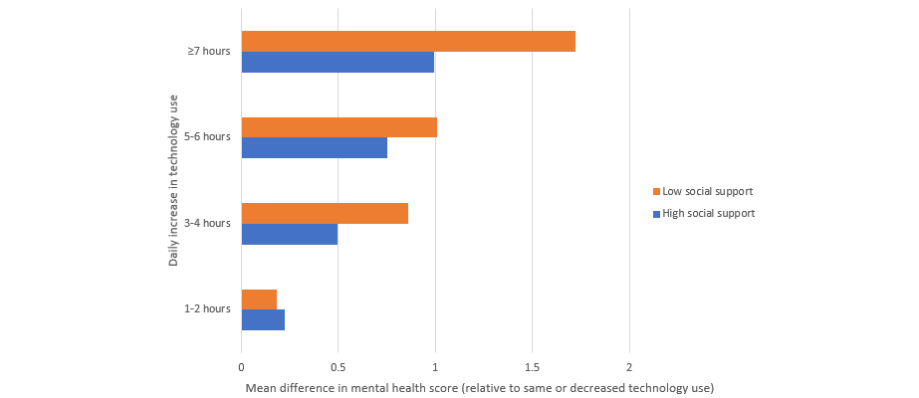
Linear regression analyses for joint effects of social support and technology use on mental health.

**Figure 2:**
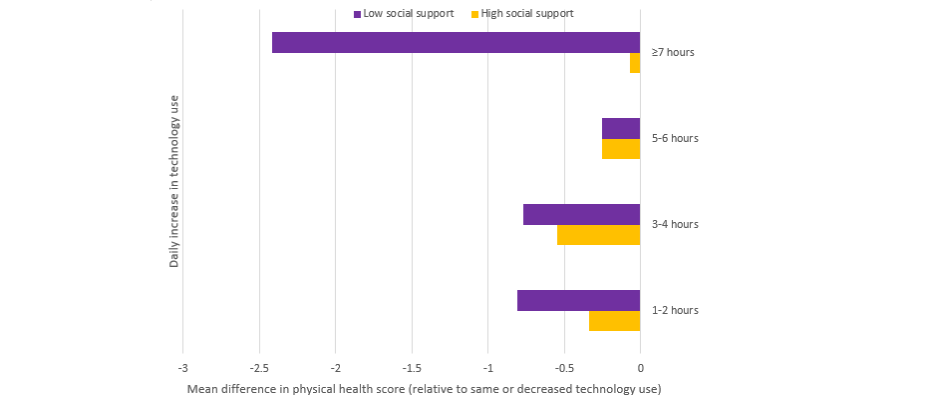
Linear regression analyses for joint effects of social support and technology use on physical health.

In exploratory analyses, we examined the joint effects of social support and technology use in relation to mental and physical health. Social support and technology use were independently associated with each other in both models, and the interaction between the two was significant for both mental and physical health. We found that the effect of technology use on mental health was significantly weaker among those with low social support compared with among those with high social support (*P*=.01; Figure 1). Similarly, the effect of technology on physical health was significantly weaker among those with low social support (*P*=.02; Figure 2). The interaction finding with social support and technology use with both mental and physical health was tested for an overall effect but was not specific to certain length of time (hours) of technology use. Although there was a significant overall interaction for the measures, they were more pronounced at higher levels.

## Discussion

### Principal Findings

This paper presents the association of self-reported social support and technology use on the outcomes of mental and physical health during the COVID-19 pandemic as reported by over 4600 AAPI community members in a national survey. We found several significant results that represent 10 different AAPI ethnic groups; however, our paper presents stratified analyses in the 3 largest ethnic groups: Ethnic Chinese, Korean, and Vietnamese. Overall, 12.9% and 11.4% of our survey respondents reported depression and anxiety, respectively, which is markedly higher than prepandemic averages. According to the 2019 US Census Bureau assessments prior to the pandemic, only 2.9% and 3.2% of Asian Americans reported depressive and anxiety disorders, respectively (n=31,229) [52]. Among all US adults, there was a 4-fold increase in the likelihood of having depression or anxiety from 2019 to 2020, with the greatest increases among younger people, Asian Americans, and parents [52]. The average physical health score of 78.1 (out of 100) among AAPIs in our study was similar to what was reported in a prior study of over 11,500 diverse adults 18 years and older (eg, score of 79.2) [53].

In this cross-sectional study, we found that low social support was associated with more self-reported mental health symptoms (depression and anxiety) overall. Furthermore, although both age groups had these outcomes, when comparing the 2 age groups in the adjusted analyses, we found all types of social support were more significant for those younger than 60 years of age compared with those 60 years and older, suggesting greater impact of social support among younger adults with more mental health symptoms. A rise in mental health changes (ie, depression, fear, health-related anxiety) would be expected given the increased social isolation due to SIP orders and social distancing, as well as increased concern for self and loved ones (eg, health, financial stability) caused by the COVID-19 pandemic [54]. Reasons for the greater mental health impact in the younger group may be related to being accustomed to more socialization compared with those who are older who may be more resilient to social changes and less socialization [55]. No significant differences were found among AAPI ethnic groups. Potential reasons why depression and anxiety were lower in adults 60 years and older compared with younger adults include the public health message that isolation was protective against COVID-19; in addition to older adults being more used to not socializing as much as younger people, they were also likely more grateful to be alone, due to their awareness of being in an at-risk group. However, the literature on older adults, not specific to AAPI, shows that they were more likely at a high risk for mental illness from social isolation in general and social restrictions due to COVID-19 [56,57].

Beyond mental health outcomes, we found that high social support was associated with positive self-reported physical health scores. After adjusting for possible confounding variables, findings on social support and physical health scores were limited to those who were less than 60 years of age, unlike social support and mental health. Again, we did not detect significant differences among ethnic groups. By examining access and reliance on all forms of social support, our results confer our prepandemic understanding of coping in which forms of social support are used to improve health status and have a protective factor. Compared with this survey, a cross-sectional study of 3649 individuals aged 60 years and older concluded that the predictors of poor self-reported health included a lack of perceived social support in women and lack of social network involvement in men [58].

With regards to technology use during the pandemic, we found incremental increases in self-reported depressive and anxiety scores with increased use of technology; mental health symptoms (ie, higher PHQ-4 scores up to 2.15) were greater with technology use of 3 or more hours after adjusting for all possible confounders. These results are consistent with other research that showed that moderate or severe levels of depression were associated with more than 2-fold time spent watching TV and using a computer (>6 hours per day) [59]. Similarly, increased technology use was associated with poor physical health. Unlike social support, there was no significant interaction with the age groups. We did not detect significant differences among cultural groups.

When examining technology use and physical health, we detected significant changes in self-reported physical health scores with 3 or more hours per day of technology use after adjusting for all possible confounders. Consistent with our results, increased screen time has been linked to potentially harmful physical effects, as it negatively affects physical activity and sleep routine, thereby leading to headaches, neck pain, myopia, digital eye syndrome, and cardiovascular risk factors (obesity, high blood pressure, and insulin resistance) [60]. Additional analyses showed differences between cultural groups, with Korean American individuals reporting lower physical health scores than ethnic Chinese and Vietnamese individuals who had the same or lower amount of technology use. Our survey did not ask participants to specify the reason for using technology, so it is unclear whether this signals more technology use for work, leisure, or both. Based on increases in internet service use (from 40% to 100%) and video-conferencing services (10-fold increase) during the pandemic, we can assume a good portion was used for work purposes across all groups [61].

### Comparisons With Prior Work

When examining the joint effects of both social support and technology use, we found significant independent associations with mental and physical health as well as a significant interaction between social support and technology use for both mental and physical health after accounting for all possible confounders. When considering technology, an important factor is social media use, with one study showing that social media exposure was associated with anxiety and combined depression and anxiety when compared with less social media exposure (1.72 and 1.91 greater odds, respectively) [12]. In terms of the interaction effect between social support and technology use on physical health, there are important potential benefits. In a study of 591 older adults, social connectedness via technology prior to the pandemic was associated with significant health benefits including better self-rated health, fewer chronic illnesses, higher subjective well-being, and fewer depressive symptoms [62].

### Clinical Implications

There are important clinical implications related to our findings, particularly for mental health providers, public health officials, and community partners. Undoubtedly, the COVID-19 pandemic negatively impacted mental and physical health for the vast majority of adults and children; however, understanding the contributing factors and high-risk groups is critical. Adults younger than 60 years were more impacted by social support, as evidenced by stronger associations with lower mental health and higher physical health scores compared with those who were 60 years or older. In addition, Korean Americans appeared to be a high-risk group for poor physical health with increased technology use. Further work is needed to understand how social support is characterized and how to best operationalize it for each AAPI community to achieve the known mental and physical health benefits in times of emergency [63]. Although access to technology may have buffered loneliness and isolation [64], more research is needed on the potential for different uses of technology to improve self-care and well-being, along with the potential to bring harm to individuals.

### Limitations

There are limitations that should be considered when interpreting these results, including a cross-sectional survey study design that did not allow us to assess dynamic changes over time during the pandemic. Another limitation was nonprobability sampling. We had significantly more female participants than male participants, although we adjusted for this in our analyses. Although this was a national survey, there were varying SIP and social distancing policies across regions and states, which may have affected the responses. Since the survey was not conducted in every AAPI language spoken, generalizability is limited and would be improved with more languages added to the survey. In addition, COMPASS is primarily an online survey, limiting its reach to those who lack access or are unable to utilize the internet. As mentioned, our inquiry on technology use did not distinguish between varying technologies nor whether the technologies were used for work or leisure.

### Conclusions

The COVID-19 pandemic has taken its toll on both mental and physical health in many individuals and families in the United States and globally. In a large sample of AAPI recruited nationally, a significant portion of our study population reported probable anxiety and depression. The prevalence of moderate and severe depressive symptoms was lower among those aged 60 years and older compared with those younger than 60 years. Despite COVID-19–related deaths being highly concentrated in this older age group, this finding represents the resilience of older AAPI Americans during a pandemic, with less than 5% of older adults reporting moderate to severe depressive symptoms.

Importantly, this study offered new insights into contributing factors for mental and physical health status by examining perceived social support and changes in technology use among AAPI during the pandemic. The key findings suggested that greater social support during the pandemic was associated with lower levels of depression and anxiety and better physical health, whereas increased technology use during the pandemic was associated with poorer mental and physical health in AAPI. Such associations were particularly pronounced among adults younger than 60 years. Although we observed similar associations between social support and mental health across cultural groups, Korean Americans appeared to be more vulnerable to the effect of increased technology use on their physical health. These findings revealed new health disparities observed during the pandemic that require urgent attention on multiple fronts, including individual, social, and community levels. Future research on how social support can be leveraged, especially among those younger than 60 years, and how various types of technology are being utilized is important to guide the recovery efforts to address both mental and physical disparities across communities as a result of the negative impacts from the COVID-19 pandemic.

## Appendix

Multimedia Appendix 1 Technology Use for Support During COVID-19 Survey.

Multimedia Appendix 2 Baseline technology use between ethnic groups.

## Abbreviations

AAPI: Asian Americans and Pacific Islander
CARE: The Collaborative Approach for Asian Americans and Pacific Islanders Research and Education
COMPASS: COVID-19 Effects on the Mental and Physical Health of Asian Americans and Pacific Islanders Survey Study
ICAN: International Children Assistance Network
NAPCA: National Asian Pacific Center on Aging
NIA: National Institute on Aging
NIH: National Institutes of Health
PHQ-4: Patient Health Questionnaire-4
SIP: shelter-in-place
VIF: variance inflation factor
