# Supplementary material for: Social Support and Technology Use and Their Association With Mental and Physical Health During the COVID-19 Pandemic Among Asian Americans: The COMPASS Cross-sectional Study
Source: JMIR Public Health Surveill. 2023 Jan 23;9:e35748. doi: 10.2196/35748 (PMC9872978; doi:10.2196/35748)
Supplement: Multimedia Appendix 1 [file publichealth_v9i1e35748_app1.docx]

**Technology Use for Support During COVID-19 Survey**

This questionnaire focuses on your use of digital technology for education and resources as well as self-care and social connection during the COVID-19 pandemic.

1. Do you own or have easy access to (check all that apply):

__ A smartphone

__ Desktop or laptop computer

__ Home internet (Wifi)

__ Tablet (iPad, e-reader, etc.)

__ Television with cable

1. As a result of the COVID-19 pandemic, I used technology for: (check all that apply):

__ Video chatting with friends or family (Zoom, WhatsApp, FaceTime, etc.)

__ Video meetings for work-related activities

__ Phone or video visit with a health care provider

__ Exercise or activities for physical fitness

__ Mental health activities

__ Keeping in touch with friends and family through social media

__ Leisure activities or hobbies

__ Accessing news

1. How many hours are you connected to technology during the day?

__ 1-4 hours/day

__ 5-8 hours/day

__ 9-12 hours/day

__ 13-16 hours/day

__ over 17+ hours/day

1. Did your use of technology increase during the COVID-19 crisis?

__ Did not increase

__ Increased by 1-2 hours/day

__ Increased by 3-4 hours/day

__ Increased by 5-6 hours/day

__ Increased by 7+ hours/day

Please state your level of agreement with each of these sentences:

1. I am comfortable with using technology and do not usually experience difficulties.
2. Not at all
3. Slightly
4. Moderately
5. Very
6. Extremely
7. Using technology was helpful for my physical health:
8. Not at all
9. Slightly
10. Moderately
11. Very
12. Extremely
13. Using technology was helpful for my mental health:
14. Not at all
15. Slightly
16. Moderately
17. Very
18. Extremely
19. Using technology was helpful for keeping up with the news:
20. Not at all
21. Slightly
22. Moderately
23. Very
24. Extremely
25. Using technology was helpful for keeping in touch for social connections:
26. Not at all
27. Slightly
28. Moderately
29. Very
30. Extremely
31. Using technology was helpful to care for others:
32. Not at all
33. Slightly
34. Moderately
35. Very
36. Extremely

11) Is there anything else you want to tell us about your use of technology during COVID-19?

____________________________________________________________________________

____________________________________________________________________________

___________________________________________________________________________
