# Supplementary material for: Social Support and Technology Use and Their Association With Mental and Physical Health During the COVID-19 Pandemic Among Asian Americans: The COMPASS Cross-sectional Study
Source: JMIR Public Health Surveill. 2023 Jan 23;9:e35748. doi: 10.2196/35748 (PMC9872978; doi:10.2196/35748)
Supplement: Multimedia Appendix 2 [file publichealth_v9i1e35748_app2.docx]

**Supplemental Table 1: Baseline Technology Use between Ethnic Groups**

**(p<.001)**

| **Baseline Daily Tech Usage** | **All** | **Asian Indian** | **Ethnic Chinese** | **Filipino** | **Hmong** | **Japanese** | **Korean** | **NHPI** | **Vietnamese** | **Other/Mixed** |
| --- | --- | --- | --- | --- | --- | --- | --- | --- | --- | --- |
| <1 hour | 136 (2.9) | 6 (2.1) | 17 (1.1) | 2 (1.2) | 1 (1.0) | 3 (1.5) | 23 (2.2) | 18 (7.3) | 62 (7.4) | 4 (1.7) |
| 1-4 hours | 1,164 (25.2) | 92 (32.6) | 319 (20.5) | 32 (19.4) | 9 (9.3) | 44 (21.9) | 289 (28.8) | 65 (26.4) | 286 (34.3) | 28 (12.1) |
| 5-8 hours | 1,406 (30.5) | 79 (28.0) | 484 (31.3) | 47 (28.5) | 29 (29.9) | 73 (36.3) | 318 (31.7) | 82 (33.3) | 218 (26.1) | 76 (32.8) |
| 9-12 hours | 1,209 (26.2) | 73 (25.9) | 475 (30.5) | 38 (23.0) | 33 (34.0) | 59 (29.4) | 241 (24.1) | 55 (22.4) | 152 (18.2) | 83 (35.8) |
| 13-16 hours | 530 (11.5) | 25 (8.9) | 208 (13.3) | 26 (15.8) | 15 (15.5) | 16 (8.0) | 105 (10.5) | 12 (4.9) | 93 (11.2) | 30 (12.9) |
| 17+ hours | 173 (3.8) | 7 (2.5) | 56 (3.6) | 20 (12.1) | 10 (10.3) | 6 (3.0) | 16 (2.6) | 14 (5.7) | 23 (2.8) | 11 (4.7) |

Data represented as n (%)
